# Supplementary material for: Simultaneous prediction of antibody backbone and side-chain conformations with deep learning
Source: PLoS One. 2022 Jun 15;17(6):e0258173. doi: 10.1371/journal.pone.0258173 (PMC9200299; doi:10.1371/journal.pone.0258173)
Supplement: S1 File — File containing containing supporting tables and figures for DeepSCAb method for prediction of antibody side-chain conformations. (PDF) [file pone.0258173.s001.pdf]

Supporting information

Supp. Table 1. Average RMSD (in Å) at relative SC SASA values for the CDR H3 loop

Relative SC SASA	Method			
	PEARS	SCWRL4	Rosetta	DeepSCAb
0	.55Å ± .83	.82Å ± .94	.72Å ± .90	.64Å ± .85
(0, 0.1]	.86Å ± 1.1	1.1Å ±1.0	.99Å ± 1.0	.94Å ± .97
(0.1, 0.2]	.89Å ± 1.1	1.2Å ±1.0	1.2Å ± 1.1	1.1Å ± 1.0
(0.2, 0.3]	.92Å ± 1.1	1.1Å ±.98	1.1Å ± 1.1	1.1Å ± 1.0
(0.3, 0.4]	1.0Å ± 1.2	1.2Å ±1.1	1.2Å ± 1.1	1.2Å ± 1.1
(0.4, 0.5]	1.4Å ± 1.3	1.5Å ±1.2	1.4Å ± 1.2	1.4Å ± 1.2
(0.5, 0.6]	1.7Å ± 1.3	1.7Å ± 1.2	1.6Å ± 1.2	1.6Å ± 1.2
(0.6, 0.7]	2.1Å ± 1.4	2.4Å ± 1.4	2.3Å ± 1.4	2.3Å ± 1.4
(0.7, 1]	2.0Å ± 1.8	2.9Å ± 1.5	2.7Å ± 1.5	2.7Å ± 1.5

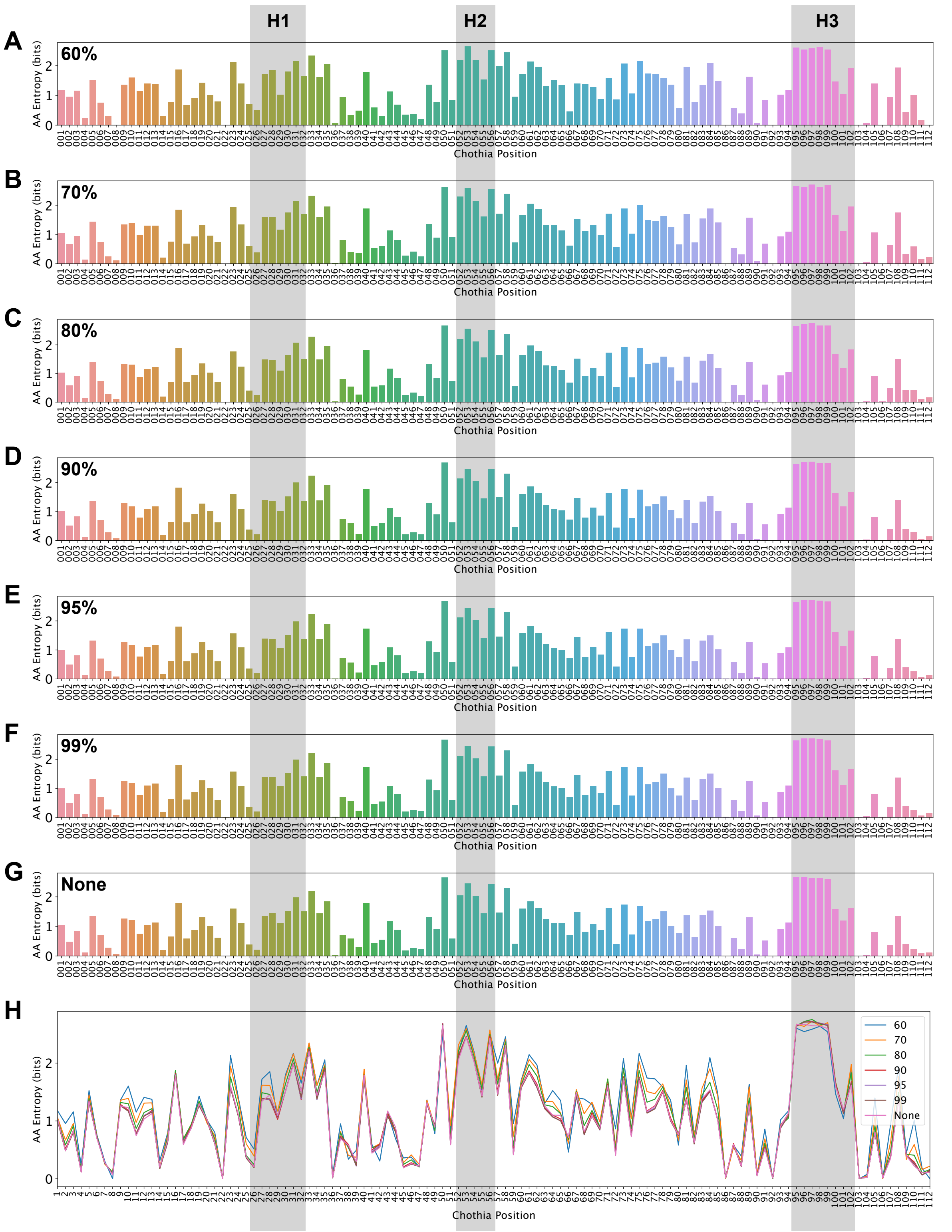

Supp. Figure 1. Impact of sequence identity cutoff on diversity of antibody heavy chain sequences. For each Chothia-numbered heavy chain position, the entropy of the distribution of amino acid sequences is calculated. For residues with insertion codes, statistics are aggregated with the primary, non-insertion, position. Residues belonging to the CDR loops are indicated by gray bands. (A-F) Positional amino acid entropy for datasets with increasing sequence identity cutoffs (60% to 99%). (G) Positional amino acid entropy for dataset with no sequence identity cutoff. (H) Comparison of positional entropy for non-redundant datasets and unfiltered dataset.

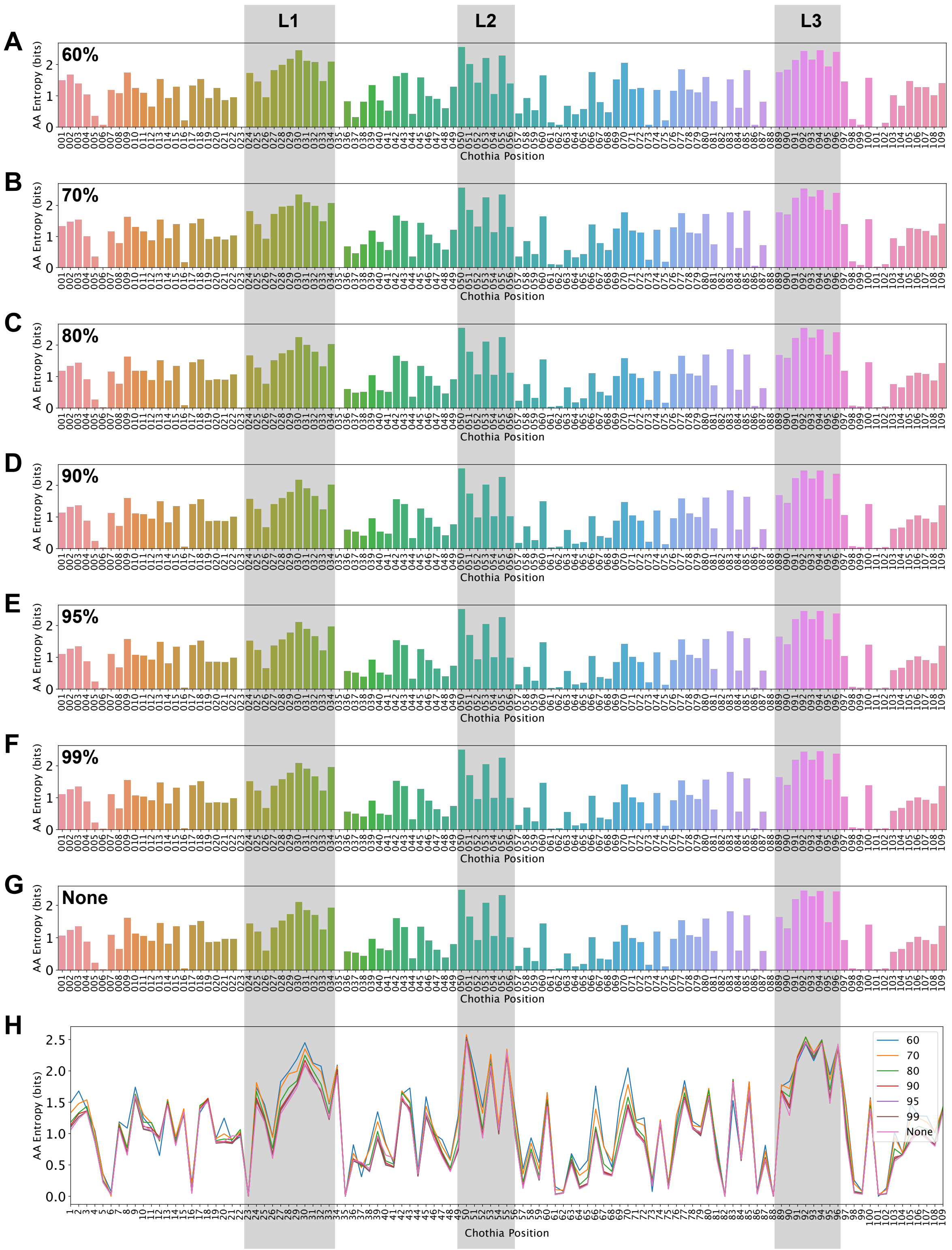

Supp. Figure 2. Impact of sequence identity cutoff on diversity of antibody light chain sequences. For each Chothia-numbered light chain position, the entropy of the distribution of amino acid sequences is calculated. For residues with insertion codes, statistics are aggregated with the primary, non-insertion, position. Residues belonging to the CDR loops are indicated by gray bands. (A-F) Positional amino acid entropy for datasets with increasing sequence identity cutoffs (60% to 99%). (G) Positional amino acid entropy for dataset with no sequence identity cutoff. (H) Comparison of positional entropy for non-redundant datasets and unfiltered dataset.

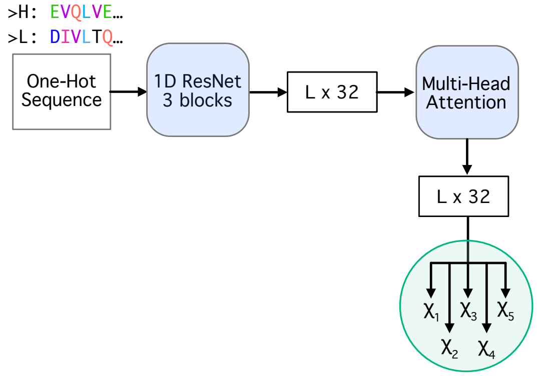

Supp. Figure 3. side-chain-only control network architecture. The control network has a similar architecture to the rotamer module in DeepSCAb.

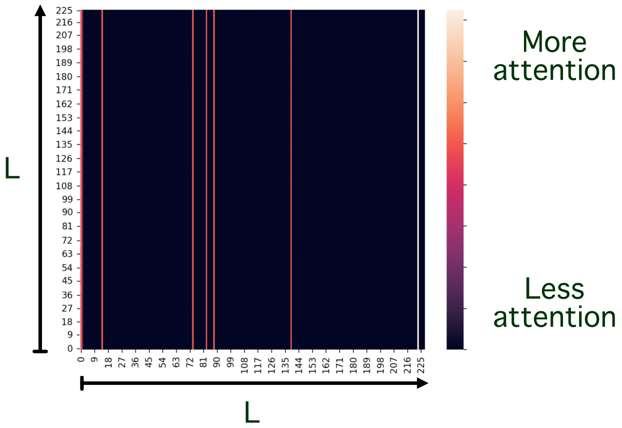

Supp. Figure 4. Interpretation of the attention matrices. L matrix is shown for a target chosen at random. The final attention is calculated by averaging over the rows to collapse the matrix to dimension $L \times 1$.

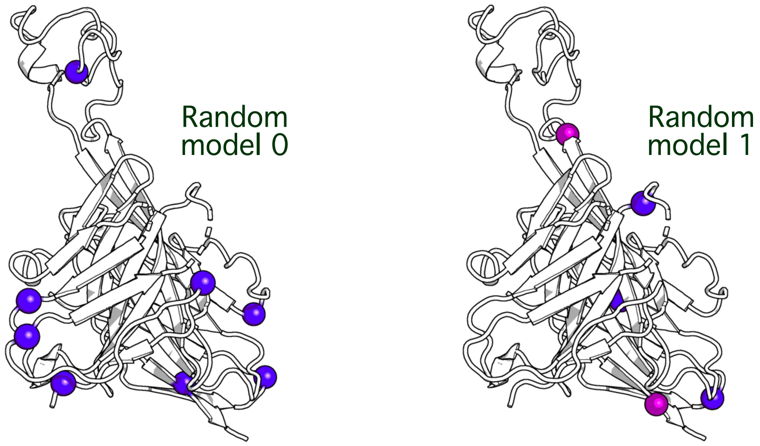

Supp. Figure 5. Comparison of anchor positions between two random models of DeepSCAb. The bovine PDB 6E9G is interpreted by two different models to show the variation of anchor positions.

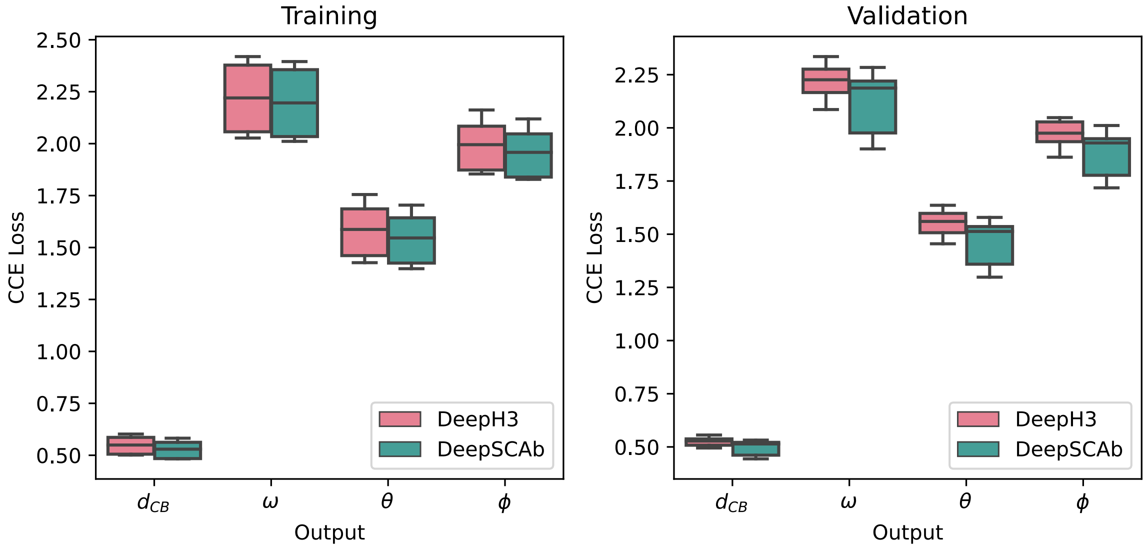

Supp. Figure 6. Comparison of pairwise geometry prediction loss for DeepH3 and DeepSCAb. Final training and validation losses achieved by DeepH3 and DeepSCAb model ensembles for each pairwise geometry output. DeepH3 is initially trained to predict only pairwise geometries (pink), then adapted into DeepSCAb to simultaneously predict pairwise geometries and side-chain conformations (teal).
