# Supplementary material for: Simultaneous prediction of antibody backbone and side-chain conformations with deep learning
Source: PLoS One. 2022 Jun 15;17(6):e0258173. doi: 10.1371/journal.pone.0258173 (PMC9200299; doi:10.1371/journal.pone.0258173)
Supplement: Attachment — Submitted filename: Response to PLOS reviewers.pdf [file pone.0258173.s002.pdf]

Response to reviewers

We thank the reviewers for their comments and critiques, which have undoubtedly resulted in a stronger manuscript. Below we detail the changes made in response to the reviewer's comments. For convenience, the original reviewer comments are included, while our responses are indented below. Changes to the manuscript are indicated by *blue text*.

<u>Academic Editor</u>

Scientific issues:

1. Methods section does not contain any information about validation/control sets.

> We have expanded our description of the structural dataset used for this work, including the following addition about the proportion used for training and validation:
>
> *Of these structures, a random 95% were used for training and the remaining 5% were used for validation.*

2. The input vector is not fully defined. What is L: the length of the entire antibody, variable region of both chains, or CDR part only? L as an input assumes a constant value, but all antibodies, especially in Fv part only, are of variable length. When a shorter sequence is considered, how the empty positions are filled, padded with 0s or something else? If the input is in one- hot format, why the chain delimiter occupies only one position, not 2? What is the input format for the 21st position (light vs heavy chain) used then?

> The input sequence length corresponds to the cumulative length of the combined heavy and light chains. Because our model is fully convolutional, the input dimension can scale arbitrarily along its length, allowing for variable length inputs. Although in this work we train with a batch size of 1 sequence, it is also common to train on more sequences simultaneously via padding. We have expanded our description of the model input to more clearly describe the length of the input, as well as the purpose of the 21 encoding dimensions at each position in the sequence:
>
> *The inter-residue module consists of a 3-block 1D ResNet and a 25-block 2D ResNet. As input to the model, we provide the concatenated heavy and light chain FV sequences, with a total length L. The input amino acid sequence is one-hot encoded, resulting in a dimension L ×20. We append an additional binary chain-break delimiter, dimension L ×1, to the input encoding to mark the last residue of the heavy chain. Taken together, the full model input has dimension L ×21.*

3. 99% sequence identity threshold appears very permissive that may result in the over-optimistic results. The authors need to compute and provide distributions of the antibodies per bins of sequence identity, using e.g. USEARCH/UCLUST tool, followed by the distribution as

to where most of those mismatches fall into for each sequence identity bin (e.g., in the CDR or otherwise).

We thank the reviewers for their suggestion of how to assess the impacts of sequence identity on dataset diversity. As suggested, we have collected structural databases from SAbDab at a range of sequence identity cutoffs (60%, 70%, 80%, 90%, 95%, 99%), as well as an unfiltered set. For each dataset, we have calculated the entropy of the amino acid distribution at each Chothia-numbered position. In general, we find that relaxing the sequence identity cutoff results in lower positional entropy. However, for the challenging CDR H3 loop, we observe greater diversity with relaxed sequence identity thresholds (but not for unfiltered structures). We believe this analysis supports our choice of 99% sequence identity for the model training dataset. We have added the following text to the manuscript, as well as two new supplemental figures (SFigure 1, SFigure 2), describing this analysis:

To assess the impacts of the sequence redundancy threshold on antibody sequence diversity, we collected structures filtered at a range of sequence identity cutoffs (60%, 70%, 80%, 90%, 95%, 99%), as well as an unfiltered set of structures. For each set of structures, we calculated the entropy of the amino acid distribution for each position according to the Chothia numbering (Figure S 1, Figure S 2). As expected, we observed a general loss of positional diversity (lower entropy) with increasing sequence redundancy. However, we observed the opposite trend for the residues belonging to the CDR H3 loop, with less stringent cutoffs allowing for greater sequence diversity. With this in mind, we selected the 99% sequence identity dataset for model training.

4. While the authors claim importance of the predictions to the subsequent AB-antigen docking, they provided no information as to how antigens and induced fit were accounted for in their model. Furthermore, the authors need to discuss and describe the following aspects of training and validating of predicted structural data: (1) Report the number of AB structures in bound and unbound forms used in training and test sets. (2) Given that many loop regions (which are important in the context of CDR) are frequently too flexible to be resolved by X-ray or result in multiple occupancies in the ATOM section of PDB file, the authors have to describe how they used structures with missing atoms or atoms with multiple occupancies. The same pertains to NMR-based modes – which model from the ensemble was used for training and validation?

We have revised the introductory paragraph of our discussion to instead focus more concretely on the ways we believe our results will improve existing methods for antibody structure prediction, rather than look forward more speculatively towards improvements in docking, as our model does not directly account for antigens:

The results outlined in our work show that our method is a step towards accurate antibody structure prediction via inclusion of side chain predictions.

The training dataset consists of 911 (64%) bound and 522 (36%) unbound antibody structures. We have added the following text to Training dataset for clarification:

…resulting in a total of 1,433 antibody structures (64% bound and 36% unbound) for training and validation of our network.

For PDBs with multiple entries, we always take the first structure. We have added this detail to the description of the training dataset construction:

For PDBs with multiple structures (such as crystals with multiple instances of the Fv in the unit cell), we always select the first.

Finally, we have added the following text to the model training section of our methods describing how we account for missing residues, while still learning from the remaining structure:

We do not calculate losses for residue pairs and rotamers missing any of their constitutive atoms, as can occur for poorly resolved flexible regions.

5. Methods should contain information how values of relative solvent accessibility were computed. Figure 4 contains ranges with SASA > 100%, which is confusing.

To calculate relative side chain SASA values, we used the Rosetta rel_per_res_sc_sasa function. After investigating further based on the reviewer's comment, we have determined an issue with this function: the ASA normalization performed by Rosetta uses an outdated table of ASA values (Miller et al. 1987). Instead of obtaining relative SASA with Rosetta, we decided to scale the SASA data based on revisited values for ASA normalization (Tien et al. 2013), which directly addressed the relative SASA percentage discrepancy. We have updated the following text providing these details.

We evaluated the performance of our method and three alternative methods as a function of relative side chain solvent accessible surface area (SC SASA) using the Rosetta rel_per_res_sc_sasa method, normalizing using the updated values from Tien, et al.

6. If not demonstrated in Results, the issue of cross-reactivity of Abs should be at least pointed out in Discussion. There are many instances, e.g. in autoimmune disorders, when the same Ab naturally evolved against viral proteins cross-reacts with the human (host in general) proteins that have no sequence similarity to the viral antigens. For example, use published reports in lupus. The authors at least need to offer some hypothesis in Discussion as to why this may happen.

The issue of cross-reactivity of antibodies is indeed an interesting area of investigation, and structure prediction may play a critical role in addressing such outstanding

questions. However, the focus of the present work is on the development of methods for antibody structure prediction and does not model the presence of the antigen. Given this, we believe that specific application to the question of cross-reactivity is beyond the scope of this work.

Editorial issues:

1. Description of the Training set appears to be copied verbatim from the authors` previous publication, which technically falls into self-plagiarism category.

We apologize and thank the editor for detecting this oversight. We have rewritten the description of the training dataset, as well as incorporated further feedback from the reviewers:

We used the Structural Antibody Database [20], SAbDab, to curate the training dataset for DeepSCAb. To ensure only high-quality examples were used for training, we limited the dataset to structures with 3 Å resolution or better. To assess the impacts of the sequence redundancy threshold on antibody sequence diversity, we collected structures filtered at a range of sequence identity cutoffs (60%, 70%, 80%, 90%, 95%, 99%), as well as an unfiltered set of structures. For each set of structures, we calculated the entropy of the amino acid distribution for each position according to the Chothia numbering (Figure S 1, Figure S 2). As expected, we observed a general loss of positional diversity (lower entropy) with increasing sequence redundancy. However, we observed the opposite trend for the residues belonging to the CDR H3 loop, with less stringent cutoffs allowing for greater sequence diversity. With this in mind, we selected the 99% sequence identity dataset for model training. For PDBs with multiple structures (such as crystals with multiple instances of the FV in the unit cell), we always select the first. We additionally removed targets belonging to the RosettaAntibody benchmark set [21] to evaluate model performance, resulting in a total of 1,433 antibody structures (64% bound and 36% unbound) for training and validation of our network. Of these structures, a random 95% were used for training and the remaining 5% were used for validation.

2. All references have to adhere to the scientific citation format. For example, references 14, 15, and 16 do not contain the source of publication, such as journal.

We have corrected the references to include the missing information.

3. All associated software should be made publicly available in order to allow reviewers to assess its functionality.

The code and data are now available at https://github.com/Graylab/DeepSCAb.

Reviewer #1

Summary:
The authors develop a ML pipeline that predicts not only antibody structure from its sequence, but also the side chain conformations. Of note, predicting antibody structure is still a very challenging task for which Alphafold2 has shown disappointing results (probably due to the lack of co-evolutionary information between antibodies and antigens). Therefore, there is critical need for such type of tools.
Particularly interesting points:
- The authors relax predicted atomic/residue distances into a realistic 3D structure using Rosetta, which is therefore more useful and makes comparison to experimental structures easier.
- This study extends their previous work (Ref 13) by additionally prediting the side chain of the antibody, which is indeed a lacking point of most predictions methods. As they write, Alphafold2 does predict side residue conformation (but with low accuracy), so a tool specifically benchmarked for antibodies is needed. Abodybuilder does predict it though.
- The authors use the attention layer as to interprete the results (which anchors are most determinant in the side chains conformations), which is a good example of gained knowledge/interpretability from a trained model, which is appreciated. - We believe the authors provided convincing sets of controls as to show the preformance of this tool.

Major comments:

Major point:

- We recall that AbodyBuilder also predicts side-chains in two ways: "complete" prediction, where every side chain is predicted (using PEARS, we think), and "partial" prediction, where side chains of identical residues from the template are retained, and the remaining side chains are also predicted. Wouldn’t it make sense to compare the performance to AbodyBuilder (and not only PEARS alone), or did we miss something?

> In order to fairly evaluate the performance of side chain prediction methods, it is important to have a ground truth solution to compare all methods. Defining the ground truth becomes difficult if we compare side chain methods on separate backbone structures, as it is not clear whether a performant method should recapitulate the native side chains or produce the best side chains given the backbone. As such, we provide all methods with the native backbone and measure the recovery of the native side chains, for which we have a “correct” answer from the crystal structure.

Minor comments:

- In case there is a revision round, please make the code and data available to the reviewers. We couldn’t assess whether it is easy to use/reproducible.

> The code and data are now available at https://github.com/Graylab/DeepSCAb.

- The language is pretty technical, and some concepts could be better explained to non-specialists, such as the interest of usnig decoy discrimination, the conditional prediction of side chains in Figure 1A.

> We have adjusted our language and added examples for the concepts the review noted, and hope that these changes will improve the accessibility of our manuscript.
>
> We have added the following text to explain the interest of using decoy discrimination for evaluating energy functions:
>
> *In the decoy discrimination task, we evaluate the ability of an energy function (such as the pairwise and rotameric distributions learned by DeepSCAb) to distinguish near-native conformations from a large set of alternative conformations (decoys).*
>
> To better explain the conditional prediction of side chains, we added the following to Simultaneous prediction of side chain and backbone geometries:
>
> *For example, χ_1 is an input to χ_2, χ_1 and χ_2 are inputs to χ_3, χ_1 through χ_3 are inputs to χ_4, andχ_1 through χ_4 are inputs to χ_5.*

- The discussion starts with the importance of the work in the context of docking, but is not really substantiated, although it is likely true... Could the authors discuss more reasons to believe so? For instance, devil's advocate could say that due to side chain flexibility, knowing the side chains might or might not help docking that much.

> We have revised the first paragraph of our discussion to instead focus more concretely on the ways we believe our results will improve existing methods for antibody structure prediction, as well as how they might forecast continued improvement in side chain prediction as backbone prediction improves:
>
> *The results outlined in our work show that our method is a step towards accurate antibody structure prediction via inclusion of side-chain conformations. We demonstrated that DeepSCAb predictions remain competitively accurate at varying side-chain surface exposure. In investigating the causes of failed side-chain predictions, we found that DeepSCAb rotamer module performance is dependent on the quality of its inter-residue geometry predictions. Thus, as methods for protein backbone prediction (and simultaneous side-chain prediction, as with AlphaFold2 [11]) continue to improve, it will be less important to predict side-chains separately. In the meantime, our method complements existing methods for antibody structure prediction.*

- We didn't understand whether the rotamer libraries were inputed, and from which data. How do the rotamers in the final predicted structures differ from the used rotamer library? Does it advocate for antibody-specific rotamer libraries?

Rather than input specialized rotamers for antibodies, we evaluated the ability of the energy function learned by DeepSCAb to identify correct rotamers using the standard Rosetta side chain packing machinery. We have added a more detailed description of the packing process for DeepSCAb:

The ConstraintSetMover in Rosetta applies these constraints onto the native pose and then the PackRotamersMover models side chain structures. We use the default Dunbrack rotamer library [27] and allow the PackRotamersMover to sample extended ranges for X1 and X2 (using the "-ex1" and "-ex2" flags), as this has been shown to improve side chain packing performance. We chose the standard ref2015 full-atom score function with a weight of 1.0 for all constraints. This protocol can repack side chains on any backbone structure with DeepSCAb predictions.

Reviewer #2

Summary:

In their paper 'Improved antibody structure prediction by deep learning of side chain conformations' authors present DeepSCAb - novel deep learning method of predicting structure of antibody's variable fragments from sequence. The authors use complicated, highly tailored for the task model that combines 1D and 2D residual convolutional blocks with multi-head attention module to predict dihedral angles for amino acid side chains.

In my opinion, the paper clearly explains the development of the method, performs several analyses of its output and compares its performance to other methods.
This work is of great importance for the development of new powerful biotherapeutics. I especially like how the authors predict side chain dihedral values conditionally.
However, I have the following concerns regarding this publication:

Major comments:

1. My major concerns relate to evaluation of the model's performance and comparisons to what the authors call 'control network' and DeepH3 (previous work by the authors).
First, I don't see how the idea behind the control network makes sense. The authors train it to predict side chain conformations from sequence without any information about backbone, which in my opinion is meaningless - it just violates the hierarchy of protein structure and clearly calls for much more advanced modeling techniques such as Alphafold, which first can internally infer backbone conformation and then predict side chains, making this task essentially equal to the full protein structure prediction. Therefore, I don't see how this network is useful as a baseline.

We use the control network as a baseline not because we are interested in side chain prediction performance in the absence of backbone information but because we are interested in investigating how much can be inferred about side chain conformation from solely the position of the residue. Although this would seem to violate the hierarchy of protein structures, because antibodies are largely conserved we can consider the tertiary fold largely invariant. Indeed, this baseline serves as a naive model that we hoped to see infer something akin to an IMGT-numbering scheme (similar to what the PEARS method achieves through careful collection of rotamer statistics at each conserved position). Surprisingly, this control performs quite poorly while PEARS performs quite well, indicating that more explicit injection of structural priors are necessary for antibody side chain prediction (as we show with the full DeepSCAb model). We have expanded the text to provide further motivation for control network, as well as additional interpretation of the results:

To assess the side-chain prediction accuracy of the model without any knowledge of backbone preferences, we designed a control network that consists primarily of the DeepSCAb rotamer module. Although the design of our control network would seem to violate the hierarchy of protein structure, in which the local tertiary environment is a critical determinant of side-chain conformation, we hoped to investigate the capacity of a ResNet model to infer side-chain conformations from sequence position alone. This control is similar in principle to the PEARS method [19], which uses positional statistics collected for IMGT-numbered positions to predict side-chain conformations.

We evaluated the control network and the full DeepSCAb on a decoy discrimination task using a set of structures generated by Jeliazkov, et al. [24], for the RosettaAntibody benchmark with 2,800 decoys per target. In the decoy discrimination task, we evaluate the ability of an energy function, such as the rotameric distributions predicted by DeepSCAb, to distinguish near-native conformations from a large set of alternative conformations (decoys). For each target in the benchmark, we score each of the decoys using the control network and DeepSCAb, and compare the decoy ranking capacity of the models by measuring the RMSD from the native for the top-1 and top-5 scoring structures. For the top-1 scoring structures, DeepSCAb (RMSD=3.2 Å) outperformed the control network (RMSD=5.0 Å) by 1.8 Å (32 better, 7 same, 10 worse). Among the top-5 scoring structures, DeepSCAb (RMSD=2.5 Å) outperformed the control network (RMSD=3.3 Å) by 0.8 Å (23 better, 11 same, 15 worse) (Table 1). Due to the considerable improvement observed in DeepSCAb over the control network, we conclude that direct injection of structural priors, through prediction of the inter-residue geometries, is beneficial for antibody side-chain predictions

Second, the performance improvement compared to DeepH3 is very modest, if present at all (the authors give dRMSDs of 0 and -0.1 angstroms). I would like to see uncertainties of all the RMSD and dRMSD values presented in the Table 1 and in corresponding parts of the text. This will allow to see if the performance improvements are statistically significant.

We agree that the improvement over DeepH3 is very modest, and have added the requested standard deviations to our decoy discrimation results (Table 1) to provide further clarity. Additionally, we have extended our comparison of DeepH3 and DeepSCAb to include an analysis of the prediction losses achieved by both methods. In a new supplementary figure (SFigure 5), we show that DeepSCAb achieves consistently lower loss than DeepH3 for all pairwise geometries. We have expanded the results to include this analysis:

Since DeepSCAb outperformed the side-chain-only control network, we next evaluated the impacts of learning side-chain conformations on pairwise residue-residue geometry predictions. First, we compared the cross-entropy loss achieved by DeepSCAb to that of DeepH3 for the trained ensembles (Figure S6). For every pairwise geometry prediction, DeepSCAb achieved lower loss than DeepH3 for both the training and validation datasets, suggesting that side-chain prediction can improve prediction of inter-residue geometries. Given this improvement, we next compared the performance of DeepSCAb to DeepH3 on the decoy discrimination task.

Despite these improvements in predictive performance, DeepSCAb did not yield significant improvements in CDR H3 loop discrimination, as the reviewer mentioned. We have expanded the discussion with the following text to give some insight into why this disconnect may occur:

It is well studied that access to backbone context improves side-chain predictions [18], which is in accordance with/supported by our results. Additionally, we show that inclusion of side-chains enables structure prediction models to more effectively predict pairwise geometries (i.e., lower loss). We found that informing the model of rotameric outputs improved the ability of our model to discriminate near-native CDR H3 loop structures. As this improvement is limited, rather than a significant overall improvement in predictions, we believe that the model is reducing its loss by more confidently predicting pairwise geometries that were already correct. Thus going forward we must consider an implementation of side chain learning that is tailored to pairwise geometries that the model is unable to predict correctly by itself.

In the introduction, the authors cite a number of works which present other methods for antibody structure prediction (e.g. ABLooper) and general protein structure prediction (e.g. Alphafold2 and RoseTTAFold). I think that the authors should use those methods and compare their performance to DeepSCAb's.

The methods mentioned in the introduction and by the reviewer are indeed useful and related to this work, but are not designed for prediction of side chain structures given a protein backbone. Because of this distinction, we believe our method (and the similarly performing alternatives) should be considered complementary to the advances in protein backbone structure prediction. However, we of course acknowledge that as protein

backbone prediction (and simultaneous side chain prediction, as with AlphaFold2) performance continues to improve, it will be less important to predict side chain conformations separately. We have therefore added the following text to the discussion:

The results show that our method is a step towards accurate antibody structure prediction via inclusion of side-chain conformations. We demonstrated that DeepSCAb predictions remain competitively accurate at varying side-chain surface exposure. In investigating the causes of failed side-chain predictions, we found that DeepSCAb rotamer module performance is dependent on the quality of its inter-residue geometry predictions. Thus, as methods for protein backbone prediction (and simultaneous side-chain prediction, as with AlphaFold2 [11]) continue to improve, it will be less important to predict side-chains separately. In the meantime, our method complements existing methods for antibody structure prediction.

2. The authors used PEARS, SCWRL4 and Rosetta for side chain prediction. I think that there is not enough detail given about settings used for Rosetta, which allows, in addition to the force field, tune the number of rotamers used as initial seed and other parameters, which significantly change performance. For example, there are settings that allow Rosetta to use more rotamers during packing. Did the authors explore that? Also, there are newer methods of side chain packing that claim to be better than the ones used by the authors.

Indeed, the Rosetta software package provides incredible flexibility for protein modeling tasks, including side chain packing. Although there may exist a configuration that could improve results, for this work we sought to benchmark the most reasonable default approach. Importantly, this includes the sampling of extended *X* angles (using the -ex1 -ex2 options), which substantially enhances the side chain packing performance of Rosetta. We have expanded our description of the side chain packing configuration for DeepSCAb:

The ConstraintSetMover in Rosetta applies these constraints onto the native pose and then the PackRotamersMover models side chain structures. We allow the PackRotamersMover to sample extended ranges for X1 and X2 (using the "-ex1" and "-ex2" flags), as this has been shown to improve side chain packing performance. We chose the standard ref2015 full-atom score function with a weight of 1.0 for all constraints. This protocol can repack side chains on any backbone structure with DeepSCAb predictions.

… and subsequently Rosetta:

Rosetta predictions were generated using the same protocol as DeepSCAb, but with only the ref2015 energy function and no learned constraints.

Minor comments:

1. Would be also interesting to discuss cases when DeepSCAb is worse then other methods. Why do you think this happens?

Indeed, it is important to evaluate the failure modes of a method to better inform future work. We have investigated two primary contributors to inaccurate side chain predictions from DeepSCAb. First, we looked at the impact of solvent exposure on the performance of DeepSCAb and the alternative methods (updated Figure 4A). Here, we see an expected decline in performance with increasing side chain solvent exposure. We have revised the results to more clearly describe this finding:

The exposure of side-chains to solvent (SC SASA) is a key determinant of whether a computational method can be expected to accurately recover the native side-chain conformation. In Figure 4A, we compared the repacking performance of DeepSCAb to alternatives and found that DeepSCAb produced competitive side-chain packing results for buried residues, or a relative SC SASA of 0, and across a range of increasing solvent exposures (Table S1). With increasing solvent exposure, we see a consisten degradation of performance for all methods. This is expected, as the side chains gain additional conformational freedom with increasing solvent exposure, making accurate predictions increasingly challenging.

Second, we considered how the lack of ground truth backbone might impact the performance of DeepSCAb. To investigate this, we generated backbones from the DeepSCAb pairwise geometries and compared backbone error (in terms of CB deviation from native) to error in side chain dihedrals. In a new panel of Figure 4 (Figure 4B), we show that backbone error is indeed a major factor in whether side chain dihedrals are accurately predicted. We have added the following text to the results describing this analysis and our findings:

A key distinction between DeepSCAb and alternative methods is that its learned side-chain potentials depend only on the antibody sequence. As a result, the predicted rotameric distributions are based on an implicit backbone learned by the inter-residue module. When this implicit model is incorrect, we expected the side-chain predictions to be less accurate as well. To test this hypothesis, we generated backbones from the DeepSCAb pairwise predictions using the structure realization procedure proposed for DeepAb [14]. Then, we quantify the error in this DeepSCAb backbone as the deviation from native of the Cβ atoms when the framework residues are aligned. After packing side-chains for these predicted backbones, we measure the cosine distance from the native dihedral for $\chi 1$-$\chi 4$ ($\chi 5$ is omitted due to limited data). We compare the backbone error to side-chain dihedral error and find that as the DeepSCAb-predicted backbone becomes less accurate (higher Cβ deviation), the side-chain dihedral errors increase (Figure 4B).

2. In the method description would be helpful to state explicitly what L is - is it the length of the full protein or just the length of the loop? I presume you model full protein, but do you think it could be interesting (and computationally less expensive) to model only part of anybody having most of it fixed?

> *L* represents the cumulative length of the concatenated heavy and light chain sequences. We have expanded the description of the model input to help clarify this:
>
> *The inter-residue module consists of a 3 block 1D ResNet and a 25 block 2D ResNet. As input to the model, we provide the concatenated heavy and light chain Fv sequences, with a total length L. The input amino acid sequence is one-hot encoded, resulting in a dimension Lx20. We append an additional binary chain-break delimiter, dimension Lx1, to the input encoding to mark the last residue of the heavy chain. Taken together, the full model input has dimension Lx21.*
>
> We agree with the reviewer that prediction of only certain portions of the antibody structure would be valuable. In the context of backbone structure prediction, this is the approach taken by ABlooper, which predicts CDR loops onto a given framework. With our model, it would also be possible to “freeze” the positions of known side chain conformations, thus allowing Rosetta to repack around those side chains using the DeepSCAb energies.

3. Error bars in Figure 4 should not reach negative RMSD values, they should be cut off at 0. I also not sure about usefulness of that figure given that the difference between all the method is insignificant.

> Indeed, the RMSD values should not be negative, which is now corrected. Additionally, we confirm that DeepSCAb does not predict side chains with significantly higher accuracy than PEARS, SCWRL, and Rosetta. However, our method predicts with competitive accuracy (as shown in Figure 4) without ever requiring the backbone structure to be inputted unlike PEARS, SCWRL, and Rosetta. This renders our method uniquely useful for side chain prediction in the absence of native backbone structure availability.

<u>Reviewer #3</u>

Summary:

The manuscript presents a method that explicitly addresses the antibody side chain modeling. Previous approaches focused on backbone modeling only, this is the first antibody modeling approach that predicts side chains. This is done elegantly by adding a new module to the antibody network that predicts backbone distances and angles for further optimization by Rosetta. The new module predicts the side chain rotamer angles depending on the prediction of backbone distances and angles.

Comments:

1. Test set cutoff of 99% can lead to very similar H3 loops in the training and test set.

> We agree with the reviewer that the choice of sequence identity cutoff is critical for ensuring a balanced dataset for model training and testing. As requested by the academic editor, we have conducted an analysis of the effect of sequence identity cutoff on the diversity at each position in the antibody sequence (SFigure 1, SFigure 2). This analysis demonstrated that the choice of 99% sequence identity in fact provides more sequence diversity for the residues of the H3 loop during training. We have added the following text to the methods describing this finding:
>
> *To assess the impacts of the sequence redundancy threshold on antibody sequence diversity, we collected structures filtered at a range of sequence identity cutoffs (60%, 70%, 80%, 90%, 95%, 99%), as well as an unfiltered set of structures. For each set of structures, we calculated the entropy of the amino acid distribution for each position according to the Chothia numbering (Figure S 1, Figure S 2). As expected, we observed a general loss of positional diversity (lower entropy) with increasing sequence redundancy. However, we observed the opposite trend for the residues belonging to the CDR H3 loop, with less stringent cutoffs allowing for greater sequence diversity. With this in mind, we selected the 99% sequence identity dataset for model training.*

2. Generation of 2,800 decoy models - how much time this takes for one antibody sequence? Can the same results be achieved with fewer decoys?

> Generation of decoy structures using RosettaAntibody is very compute-intensive (~20 CPU minutes per decoy). Indeed, this highlights the critical need for faster methods for antibody structure prediction. For the present study, we have reused decoy structures previously generated in order to compare the structure-scoring capabilities (i.e., decoy discrimination) for two models. However, in practice it would not be necessary to generate such decoys in order to use DeepSCAb, as only a single backbone structure is required. We have edited the following text to emphasize that the structures were generated as part of prior work, and only used here to compare the models scoring capabilities:
>
> *We evaluated the control network and the full DeepSCAb on a decoy discrimination task using a set of structures generated by Jeliazkov, et al. [23], for the RosettaAntibody benchmark (2,800 decoys per target). In the decoy discrimination task, we evaluate the ability of an energy function (such as the pairwise and rotameric distributions learned by DeepSCAb) to distinguish near-native conformations from a large set of alternative conformations (decoys). For each target in the benchmark, we score each of the decoys using the control network and DeepSCAb, and compare the decoy ranking capacity of*

the models by measuring the RMSD from the native for the top-1 and top-5 scoring structures.

3. Stated that "the addition of side chain orientations is beneficial for accurately predicting pairwise geometries." but there is no significant improvement from DeepH3 in Backbone RMSD (top1 and top 5).

We have provided further analysis to substantiate this claim by comparing the losses achieved by DeepH3 (only pairwise geometry training) and DeepSCAb (pairwise and side chain training). In a new supplementary figure (SFigure 5) we show that DeepSCAb achieves consistently lower loss than DeepH3 for all pairwise geometries. We have expanded the results to include this analysis:

Since DeepSCAb outperformed the side-chain-only control network, we next evaluated the impacts of learning side chain conformations on pairwise geometry predictions. First, we compared the cross-entropy loss achieved by DeepSCAb to that of DeepH3 for the trained ensembles (SFigure 5). For every pairwise geometry prediction, DeepSCAb achieved lower loss than DeepH3 for both the training and validation datasets, suggesting that side chain prediction can improve prediction of inter-residue geometries. Given this improvement, we next compared the performance of DeepSCAb to DeepH3 on the decoy discrimination task…

A surprising result of the present work is that improved learning of pairwise geometries did not yield significant improvements in CDR H3 loop discrimination, as discussed in the results. We have expanded the discussion with the following text to give some insight into why this disconnect may occur:

It is well studied that access to backbone context improves side-chain predictions [18], which is in accordance with/supported by our results. Additionally, we show that inclusion of side-chains enables structure prediction models to more effectively predict pairwise geometries (i.e., lower loss). We found that informing the model of rotameric outputs improved the ability of our model to discriminate near-native CDR H3 loop structures. As this improvement is limited, rather than a significant overall improvement in predictions, we believe that the model is reducing its loss by more confidently predicting pairwise geometries that were already correct. Thus going forward we must consider an implementation of side chain learning that is tailored to pairwise geometries that the model is unable to predict correctly by itself.
